# Gsk3β and Tomm20 are substrates of the SCFFbxo7/PARK15 ubiquitin ligase associated with Parkinson's disease

**DOI:** 10.1042/BCJ20160387

**Published:** 2016-10-11

**Authors:** Felipe Roberti Teixeira, Suzanne J. Randle, Shachi P. Patel, Tycho E.T. Mevissen, Grasilda Zenkeviciute, Tie Koide, David Komander, Heike Laman

**Affiliations:** 1 Department of Genetics and Evolution, Federal University of São Carlos, São Carlos, Brazil; 2 Department of Pathology, University of Cambridge, Tennis Court Road, Cambridge CB2 1QP, U.K.; 3 Department of Biochemistry and Immunology, Ribeirão Preto Medical School, University of São Paulo, Ribeirão Preto, Brazil; 4 MRC Laboratory of Molecular Biology, Francis Crick Avenue, Cambridge Biomedical Campus, Cambridge CB2 0QH, U.K.

**Keywords:** Fbxo7/PARK15, glycogen synthase kinase, mitophagy, protein array, Tomm20, ubiquitin ligases

## Abstract

Fbxo7 is a clinically relevant F-box protein, associated with both cancer and Parkinson's disease (PD). Additionally, SNPs within *FBXO7* are correlated with alterations in red blood cell parameters. Point mutations within *FBXO7* map within specific functional domains, including near its F-box domain and its substrate recruiting domains, suggesting that deficiencies in SCF^Fbxo7/PARK15^ ubiquitin ligase activity are mechanistically linked to early-onset PD. To date, relatively few substrates of the ligase have been identified. These include HURP (hepatoma up-regulated protein), whose ubiquitination results in proteasome-mediated degradation, and c-IAP1 (inhibitor of apoptosis protein 1), TNF receptor-associated factor 2 (TRAF2), and NRAGE, which are not destabilized as a result of ubiquitination. None of these substrates have been linked directly to PD, nor has it been determined whether they would directly engage neuronal cell death pathways. To discover ubiquitinated substrates of SCF^Fbxo7^ implicated more directly in PD aetiology, we conducted a high-throughput screen using protein arrays to identify new candidates. A total of 338 new targets were identified and from these we validated glycogen synthase kinase 3β (Gsk3β), which can phosphorylate α-synuclein, and translocase of outer mitochondrial membrane 20 (Tomm20), a mitochondrial translocase that, when ubiquitinated, promotes mitophagy, as SCF^Fbxo7^ substrates both *in vitro* and *in vivo*. Ubiquitin chain restriction analyses revealed that Fbxo7 modified Gsk3β using K63 linkages. Our results indicate that Fbxo7 negatively regulates Gsk3β activity, rather than its levels or localization. In addition, Fbxo7 ubiquitinated Tomm20, and its levels correlated with Fbxo7 expression, indicating a stabilizing effect. None of the PD-associated mutations in Fbxo7 impaired Tomm20 ubiquitination. Our findings demonstrate that SCF^Fbxo7^ has an impact directly on two proteins implicated in pathological processes leading to PD.

## Introduction

F-box proteins assemble with Skp1, Cullin 1 and Rbx1 to form SCF-type E3 ubiquitin ligases [1–3]. These enzymes act in a cascade with a ubiquitin-activating enzyme (E1) and a ubiquitin-carrier enzyme (E2) to mediate the ubiquitination of their substrates, the consequences of which range from promoting proteasomal degradation to changing the localization or activity of the modified protein. Individual substrates are thought to be targeted for ubiquitination by E3 ligases by factors such as post-translational modifications (PTMs), such as phosphorylation, glycosylation, or the phase of the cell cycle and cellular localization of the substrate and/or the ligase [4]. The biological importance of F-box proteins stems from their involvement in the regulation of many core processes such as cell cycle (Fbxl1, Fbxw5, Fbxw7, and Fbxw1), differentiation and development (Fbxw1, Fbxl14, Fbxo11, Fbxo32, and Fbxw7), cell death (Fbxw1 and Fbxw7), intracellular signaling (Fbxo25), and others (reviewed in ref. [5–8]).

Fbxo7 is a multi-functional F-box protein, with both SCF-dependent and -independent functions that are capable of impacting on many core cellular processes, and is associated with human diseases (reviewed in ref. [9]). For example, the effects of Fbxo7 on the cell cycle are independent of its ubiquitin ligase activity and are mediated instead through its interactions with cell cycle proteins, Cdk6 and p27 [10,11]. Remarkably, Fbxo7's functional effects on cell proliferation and transformation, for example, are dependent on cell type. For example, Fbxo7 overexpression transforms immortalized fibroblasts and imparts tumorigenic properties to p53 null hematopoietic stem and progenitor cells [10,12]. In contrast, in pro-B cells and pro-erythroblasts, Fbxo7 negatively regulates cell proliferation and differentiation [11]. Within other cell types, altered Fbxo7 activity may be linked to pathological changes. For example, four studies reported on many SNPs in *FBXO7* that correlated with changes in human red blood cell traits such as mean cell volume and mean cell hemoglobin, which are associated with adverse health outcomes such as anemia, cardiovascular diseases, and cancer [13–16].

Recessive mutations in *FBXO7*, also designated *PARK15*, have been identified in patients with phenotypes of idiopathic Parkinson's disease (PD) and an early-onset form of PD [17–20]. Pathological point mutations are located within specific domains which hint at Fbxo7 function(s) that might be compromised. In addition to its F-box domain, which enables incorporation into an SCF complex via Skp1 binding, Fbxo7 contains an Ubl (ubiquitin-like) domain (aa 1–79), which interacts with Parkin and PINK1; a 40 amino acid linker (aa 129–169), which binds to Cdk6; a dimerization domain (aa 182–325), which enables homo-dimerization and hetero-dimerization with PI31; and a PRR (proline-rich region) (aa 423–522), which mediates protein–protein interactions with hepatoma up-regulated protein (HURP) and Cdk6 (reviewed in ref. [9]). One PD-associated mutation, T22M, lies within the N-terminal Ubl domain and compromises its ability to interact with Parkin and to promote mitophagy [21]. A second missense mutation R378G abuts the F-box domain and compromises its ability to interact with Skp1 [22]. Finally, a point mutation R498X within the PRR truncates the final 24 aa [17]. The loss of interaction with Skp1 and a substrate-interacting domain resulting from the R378G and R498X mutations suggests that SCF^Fbxo7^-mediated ubiquitination is compromised in PD patients. However, the identity of critical targets and the functional effect of their ubiquitination are not known.

To date, there are only a handful of ubiquitinated proteins reported for SCF^Fbxo7^. These include HURP/DLG7 (hepatoma up-regulated protein), a regulator of mitotic spindle assembly, which is up-regulated in hepatocellular carcinoma, colon cancer, breast cancer, and transitional cell carcinoma [23,24]. SCF^Fbxo7^ also promotes ubiquitination of c-IAP1 (inhibitor of apoptosis) and TRAF2 resulting in decreased NF-κB signaling activity [25,26]. Most recently, NRAGE (neurotrophin receptor-interacting MAGE) was reported to be ubiquitinated by SCF^Fbxo7^, which positively regulated bone morphogenic protein (BMP) signaling [27]. Interestingly, Fbxo7 increased formation of a BMP receptor–NRAGE–TAK1–TAB1 complex and up-regulated NF-κB activity. The contrasting effects of Fbxo7 expression on NF-κB signaling may reflect cell-type-specific effects of Fbxo7 on this pathway, or alternatively, reflect the influence of other signaling inputs, like cellular stresses. There is a potential relationship between NF-κB signaling, long-term neuroinflammation and PD, as suggested, for example, by the symptoms demonstrated by the c-Rel knockout mouse [28]; however, evidence for the role of NF-κB signaling in PD remains controversial (as reviewed in ref. [29]).

There are currently no studies that define ubiquitination substrates for SCF^Fbxo7^ at a proteome-wide scale, which would greatly assist in clarifying its function(s) in its multiple clinically relevant settings. Given the contrasting biological effects of Fbxo7 expression in different cellular environments, we sought to bypass considerations of cell-type specificity, cell cycle phase, or subcellular localization, which might limit the interaction of Fbxo7 with its substrates. We undertook an *in vitro* high-throughput experimental approach utilizing a human protein microarray that displays ∼9500 individual proteins on a slide, to identify mammalian substrates for ubiquitination by SCF^Fbxo7^. This powerful approach has been used to identify substrates for ubiquitin ligases, such as yeast Rsp5 [30] and human NEDD4/NEDD4L [31], SCF^Fbxo25^ [32], and SMURF1 [33]. Our *in vitro* ubiquitination screen identified 338 unique, high-confidence Fbxo7 substrates distributed across different cellular compartments, and which are involved in many biological processes. To validate this screen, we assessed individually the *in vitro* and *in vivo* ubiquitination of two candidate proteins, Gsk3β (glycogen synthase kinase 3β) and Tomm20 (translocase of outer mitochondrial membrane 20). In addition, the type of ubiquitin chain linkages introduced by Fbxo7 onto these substrates was investigated using ubiquitin chain restriction analysis, [34] and the functional effects of their ubiquitination were tested.

## Experimental materials and methods

### Purification of SCF complexes

The SCF components such as human influenza hemagglutinin (HA)-Skp1, Cul1, Myc-Rbx1, and FLAG-Fbxo7 or FLAG-Fbxo7(ΔF-box) were transfected in HEK293T cells by using polyethylenimine. After 48 h of transfection, the cells were harvested and resuspended in lysis buffer (LB) (25 mM Tris–HCl, pH 7.5, 225 mM KCl and 1% NP-40) containing a protease inhibitor cocktail (Sigma-Aldrich, St. Louis, MO) and phosphatase inhibitors (10 mM NaF and 1 mM Na_3_VO_4_). The lysates were incubated with agarose anti-FLAG M2 beads (Sigma-Aldrich, St. Louis, MO) for 6 h at 4°C with rocking. Beads were washed with LB and the SCF complexes eluted with FLAG elution buffer (300 µg/ml of peptide FLAG in 10 mM HEPES pH 7.9, 225 mM KCl, 1.5 mM MgCl_2_, and 0.1% NP-40) for 1 h at 4°C with rocking. The eluates were stored in 15% glycerol at −20°C until use. To evaluate the purification of SCF complexes, immunoblotting was performed and probed using anti-Fbxo7 (ABN1038, Merck Millipore, Watford, UK), anti-HA (Abcam, Cambridge, UK), anti-Gsk3β (Santa Cruz Biotechnologies, CA, USA), anti-Tomm20 (Abcam, Cambridge, UK), or anti-myc (Cell Signaling Technologies, MA, USA). The concentration of the complexes was determined against known concentrations of BSA by Coomassie blue staining of the gel. The densitometry of the bands was determined by ImageJ.

### *In vitro* ubiquitination assays

The plasmids encoding human Gsk3β-HA and Tomm20 were purchased from Addgene (14 753 and 40 291, respectively). Human cIAP-1-myc was kindly provided by Dr Yasuko Matsuzawa (Sanford-Burnhan Medical Research Institute, La Jolla, CA, USA). Tomm20 was cloned into pcDNA3 in fusion with HA at the C-terminus. cIAP was truncated (183–570) and cloned in fusion with HA at the C-terminus. The substrates cIAP-1(183–570)-HA, Gsk3β-HA, and Tomm20-HA were produced by *in vitro* transcription/translation (IVT), and the crude programmed reticulocyte lysates were added to *in vitro* ubiquitination reactions. For the ubiquitination reactions, purified SCF complexes were used at the indicated concentrations in combination with ubiquitin mix [ubiquitination buffer, E1(100 nM), E2(500 nM), biotin-ubiquitin (20 µM), Mg-ATP (2 mM; Boston Biochem)], and the purified substrates and incubated for 90 min at 30°C. Proteins were resolved by SDS–PAGE and immunoblotting was performed using anti-Gsk3β, anti-Tomm20, or anti-HA antibodies. To determine which E2(s) enabled SCF^Fbxo7^ ligase activity, an *in vitro* E2 screening with 10 different E2 enzymes, each at 500 nM was performed. Auto-ubiquitination was observed with UBE2D1 (UbcH5a), UBE2D2 (UbcH5b), and UBE2D3 (UbcH5c; Supplementary Figure S1), and UBE2D1 (UbcH5a) was used in further experiments. Reactions were resolved by SDS–PAGE and detected by probing with streptavidin–HRP (Thermo Pierce, MA, USA).

### Analysis of ligases

Samples were resolved ∼1 cm into a pre-cast SDS–polyacrylamide gel, and the entire lane was excised and cut into four equal slices. Proteins were reduced and alkylated, then digested in-gel using trypsin. The resulting peptides were analyzed by LC–MS/MS using an Orbitrap XL (Thermo) coupled to a nanoAcquity UPLC (Waters). Data were acquired in a DDA fashion with MS/MS in the LTQ triggered at 1000 counts. Raw files were converted into mzML using MSconvert (ProteoWizard) and submitted to MASCOT 2.3.0 to search a human Uniprot database (20 264 entries, downloaded on 09 June 14). Carbamidomethyl cysteine was set as a fixed modification with oxidized methionine and deamidation of asparagine and glutamine as potential variable modifications. Peptide and protein validation were performed using Scaffold 4.3.2. Peptides required a minimum of 95% probability and proteins required a minimum of 90% probability and two peptides in order to be counted.

### *In vitro* ubiquitination assays of protein arrays

Protoarray® v5.0 was obtained from Life Technologies (catalog number PAH0525101). Supplementary Table S1 contains an Excel file of Protoarray® v5.0 protein content. Protocols were followed according to the manufacturer's instructions (Protoarray® v5.0, Invitrogen, MA, USA). Slides were incubated in Protoarray® Synthetic Block for 1 h at 4°C with shaking at 50 rpm. During this time, reactions were prepared in a volume of 120 µl as follows: 25 or 50 nM of the purified SCF^Fbxo7^ or Fbxo7(ΔF-box) in combination with ubiquitin mix [E1 (100 nM), UbcH5a (500 nM), Mg-ATP (2 mM), and biotin-ubiquitin 0.1 mg/ml in ubiquitination buffer; Boston Biochem]. The slides were washed with assay buffer (AB; 50 mM Tris, pH 7.5, 50 mM NaCl, 5 mM MgSO_4_, 0.1% Tween 20, 1% BSA, and 1 mM DTT) and 110 µl of the reaction was added to the slide and overlaid with a coverslip followed by incubation for 1.5 h at 30°C in a humidified chamber. Slides were washed in 0.5% SDS and AB and then incubated with 1 µg/µl of streptavidin–AlexaFluor 647 for 45 min at 4°C with shaking. The arrays were washed with AB, once with distilled water, and finally dried by centrifugation at 1000 × ***g*** for 2 min, before being scanned on a GenePix Personal 4100A (Axon–Molecular Devices).

### Data acquisition and analysis

Software used for Protoarray® image acquisition was GenePix Pro 4.1 (Molecular Devices). The experimental design comprised two biological replicates [25 or 50 nM ligase of wild type (WT) or mutant F-box protein, ΔF] with two intraslide technical replicates. Each slide has 3004 negative control spots (Supplementary Table S2) and 576 positive control spots (Supplementary Table S3).

The intensity value of each array feature was considered as the average raw intensity of all pixels in the delimited spot region minus the median intensity of pixels immediately surrounding the spot region (local background). Background-subtracted intensities were subjected to normalization to make them directly comparable among different Protoarray® slides (replicates and conditions). Assuming that the manufacturer-produced positive controls should present the same theoretical intensities among replicates, their background-corrected values were obtained and a single centering (normalization) value per slide was defined as the average of all known positive control spots. An interslide, study-wide overall positive control value was obtained by averaging all the intraslide centering/normalization factors. Finally, all array features had their background-subtracted intensities corrected by this overall factor to end up with comparable normalized intensities (*I*_WT,25 nM_, *I*_WT,50 nM_, *I*_ΔF,25 nM_, and *I*_ΔF,50 nM_).

To determine spots with statistically significant signals, we used all the negative control spots to estimate non-parametrically the null density distribution (*NC*) for each slide using a Gaussian kernel density estimator [35]. The intensity value that encompasses the vast majority of null probability mass was chosen as cutoff (*c*) for each slide: **P**(*NC* > *c*) = 0.005. This yielded four cutoff values: *c*_WT,25 nM_; *c*_WT,50 nM_; *c*_ΔF,25 nM_, and *c*_ΔF,50 nM_.

Selected spots presented intensities *I*_WT_ > *c*_WT_ and *I*_ΔF_ < *c*_ΔF_ for each concentration and log fold change [FC = log_2_(*I*_WT_/*I*_ΔF_)] greater than 5-fold: log_2_(*I*_WT_/*I*_ΔF_) > log_2_(5). To increase stringency, only proteins which met these criteria simultaneously on both concentrations were defined as possible substrates. Mean FC values of possible substrates were calculated and used to rank the proteins (Supplementary Table S4).

The selected list of proteins was subjected to Gene Enrichment analysis using DAVID Bioinformatics Resources v6.7 [36] using Protoarray® v5.0 content (Supplementary Table S1) or the human proteome as the background. DAVID's *P*-values up to 0.05 were considered significant (Supplementary Table S4).

### *In vivo* ubiquitination assays and co-immunoprecipitation assays

HEK293T cells were transfected with empty vector, FLAG-Fbxo7 or FLAG-Fbxo7-ΔF-box, or truncated FLAG-Fbxo7 alleles where indicated, in combination with Gsk3β-HA or Tomm20-HA, with or without ubiquitin-6xHis-myc. Where indicated, cells were treated with 10 µM of MG132 prior to lysis with LB for 30 min on ice. Cells were centrifuged and supernatants were subjected to immunoprecipitation (IP) with agarose-anti-HA or agarose-anti-FLAG. The proteins were eluted by Laemmli buffer and the eluates were resolved by SDS–PAGE. The ubiquitinated proteins were visualized using antibodies to HA, the substrates, or anti-myc antibody.

### *In vitro* binding assays

HEK293T cells were transfected with empty vector or various FLAG-Fbxo7 truncation mutants. Cells were lysed in NETN lysis buffer [NB; 10 mM Tris–HCl, pH 7.5, 150 mM NaCl, 1 mM EDTA, and 0.2% NP-40 containing a protease inhibitor cocktail and phosphatase inhibitors (10 mM NaF and 1 mM Na_3_VO_4_)], and clarified lysates were subjected to IP with anti-FLAG antibodies immobilized on agarose beads. Gsk3β-HA protein was IP from transfected HEK293T cells by anti-HA antibodies immobilized on agarose beads and eluted using HA peptide. Gsk3β-HA protein was added to immobilized FLAG-Fbxo7 proteins and rotated for 3 h at 4°C. Beads were washed twice in NB and twice in RIPA buffer, then eluted by Laemmli buffer and the eluates resolved by SDS–PAGE.

### Fbxo7 protein knockdown

Cells were infected with miR30-based retroviruses encoding independent human *FBXO7*-specific shRNAs and selected using puromycin (2 μg/ml), as described previously [11]. Transient knockdown (KD) of Fbxo7 was performed by transfecting dsRNA targeting Fbxo7 into cells using Effectene (Invitrogen) as described previously [10].

### Gsk3β activity reporter

The Gsk3β reporter pCS2-GFP-Gsk3-MAPK and mutant version pCS2-GFP-Gsk3mut-MAPK described in ref. [37] were purchased from Addgene (29 689 and 29 690, respectively). U2OS cells were transfected with empty vector, FLAG-Fbxo7, or FLAG-Fbxo7(ΔF-box) and 24 h later transfected with pCS2-GFP-Gsk3-MAPK or pCS2-GFP-Gsk3mut-MAPK, in quadruplicate. After a further 24 h, cells were trypsinized and analyzed by flow cytometry using a Cytek analyzer. The GFP mean fluorescence intensity was determined and levels were expressed as a percentage of the mutant GFP-Gsk3mut-MAPK-expressing cells.

### Ubiquitin chain restriction analysis

Ubiquitin chain restriction (UbiCRest) analyses were performed as described recently [34]. *In vivo* expressed substrates were IP from HEK293T cells transfected with FLAG-Fbxo7 and the tagged-HA substrates using agarose-anti-HA. Cell lysates were obtained as mentioned above and the *in vivo* polyubiquitinated substrates were eluted by HA peptide at 300 µg/ml in FLAG elution buffer and stored at −80°C. Deubiquitinating enzymes (DUBs) were diluted with 2× dilution buffer (50 mM Tris, pH 7.4, 300 mM NaCl, and 20 mM DTT) and added to the samples for 30 min at 37°C, and reactions were stopped by Laemmli buffer. Samples were run on SDS–PAGE, and the blots were probed for anti-polyubiquitin (Santa Cruz Biotechnologies, CA, USA).

### Immunofluorescence

U2OS cells were plated onto glass coverslips, transfected with FLAG-Fbxo7, and 24 h later fixed in 4% paraformaldehyde in PBS for 15 min. Cells were permeabilized in 0.2% Triton X-100 in PBS, blocked for 1 h in 10% donkey serum, and incubated overnight with anti-FLAG and anti-Gsk3β antibodies. Cells were repeatedly washed in PBS, incubated with anti-rabbit AlexaFluor 488 and anti-mouse AlexaFluor 647 for 1 h, washed again, and then counterstained with Hoechst 33342 to visualize nuclei. Cells were visualized using a Zeiss ApoTome microscope.

### Luciferase assays

U20S cells, seeded at 5000 cells per well of a 96-well plate, were transfected in quintuplicate with 1.5 ng pCMV-Renilla luciferase control plasmid, 75 ng minimal promotor firefly luciferase (pTA-luc) or LEF/TCF responsive firefly luciferase reporters (TOP-FLASH), and 75 ng empty, FLAG-Fbxo7 or FLAG-Fbxo7(ΔF-box) vectors. Where stated, cells were treated after 24 h with 50 mM LiCl or NaCl and 24 h later, luciferase levels were assayed using the Dual Glo luciferase assay system (Promega, Southampton, UK).

### Statistical analysis

Statistical differences in normalized protein levels in shRNA lines were compared with vector control levels using Student's *t*-tests. For Gsk3β reporter assays, normalized data were analyzed by one-way ANOVA, with a *post hoc* Dunnett's multiple comparisons test to determine statistical significance compared with control cells. For luciferase assays, one-way ANOVA with a *post hoc* Tukey's multiple comparisons test was used to determine differences between samples.

## Results

### Purified SCF^Fbxo7^ complexes are active *in vitro*

**Figure 1.**
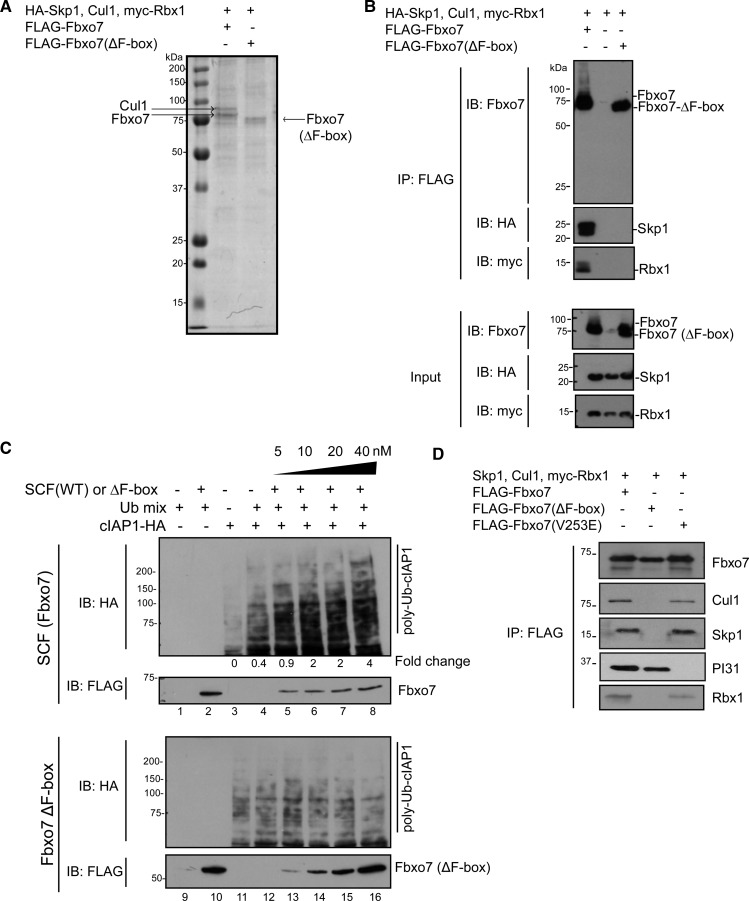
Fbxo7, but not a mutant version Fbxo7(ΔF-box), forms an active SCF complex. (**A**) Purified SCF ligases resolved by SDS–PAGE and visualized with Coomassie brilliant blue stain (*n* = 3). (**B**) Immunoblots for components of SCF holoenzyme that co-immunoprecipitate with FLAG-Fbxo7 or FLAG-Fbxo7-ΔF-box (*n* = 2). (**C**) Titration of the ligase activity of purified SCF^Fbxo7^ complexes or mutant Fbxo7(ΔF-box) protein on cIAP-1(183–570)-HA. A concentration gradient (5, 10, 20, and 40 nM) of SCF^Fbxo7^ or Fbxo7(ΔF-box) protein was used for *in vitro* ubiquitination assays in combination with ubiquitin mix (ubiquitin buffer, E1, UBE2D1, and ATP) and purified cIAP-1(183–570). Membranes were probed with anti-HA antibodies to visualize the ubiquitination profile and anti-FLAG antibodies to evaluate the amount of E3 ligase (*n* = 2). (**D**) Immunoblot for proteins that co-immunoprecipitate with FLAG-tagged SCF Fbxo7 ligases (WT, ΔF-box, or V253E).

SCF complexes were purified from mammalian HEK293T cells using mild conditions to maintain activity and structure. WT Fbxo7, but not a mutant with a deletion of the F-box domain (denoted ΔF-box, lacking amino acids 335–367), co-purified with other SCF components: Skp1, Cullin1, and Rbx1 (Figure 1A,B). To test for ligase activity, we performed *in vitro* ubiquitination assays using SCF^Fbxo7^ or the mutant Fbxo7(ΔF-box) in conjunction with both E1 and E2 enzymes. We found UBE2D1 (UbcH5a), UBE2D2 (UbcH5b), UBE2D3 (UbcH5c), and UBE2E1 (UbcH6), all promoted robust levels of auto-ubiquitination in a screen of E2 enzymes (Supplementary Figure S1A,B). To test the ability of SCF^Fbxo7^ to ubiquitinate one of its known substrates, we used cIAP-1 [25,26]. This protein contains a RING box domain that can bind independently to E2 enzymes, promoting auto-ubiquitination. As this would obscure the ubiquitination signal generated by SCF^Fbxo7^, this RING domain was deleted, creating cIAP-1(183–570)-HA, for these assays. cIAP-1(183–570)-HA was synthesized by IVT using reticulocyte lysates, and the programmed lysates were added to *in vitro* ubiquitination reactions. A smear of higher molecular weight (MW) ubiquitinated species of cIAP-1, which intensified with increasing concentration of ligase, was seen in the presence of the WT SCF^Fbxo7^, but not mutant Fbxo7(ΔF-box; Figure 1C). These experiments established that the purified SCF^Fbxo7^ ligase showed robust ligase activity and could ubiquitinate itself and one of its known substrates.

In addition to probing for known subunits of an SCF-type E3 ligase (Figure 1B), we also conducted an unbiased mass spectrometry (MS)-based screen on the purified WT ligase and the mutant Fbxo7(ΔF-box). Alongside expected SCF components, NEDD8, two proteasome subunits (PSMB3 and PSMB6), EIF4A2, FBL, HDLBP, and DNAJB6, appeared exclusively associated with SCF^Fbxo7^ and not the vector control or the mutant F-box protein made from transfection with Fbxo7(ΔF-box; Supplementary Table S4). A longer list of 107 proteins that interacted with both the WT SCF^Fbxo7^ ligases and the mutant Fbxo7 protein, included the proteasome inhibitor, PI31, a previously identified dimerization partner of Fbxo7 [38]. The association of PI31 with the ligases was confirmed by immunoblotting, where an Fbxo7(V253E) mutant that prevents binding to PI31 served as a negative control (Figure 1D). Analysis of the longer list using KEGG databases for data enrichment identified proteins from the ribosome, proteasome, and spliceosome as being significantly represented (Supplementary Table S4).

### Identification of novel SCF^Fbxo7^ substrates using a protein array

**Figure 2.**
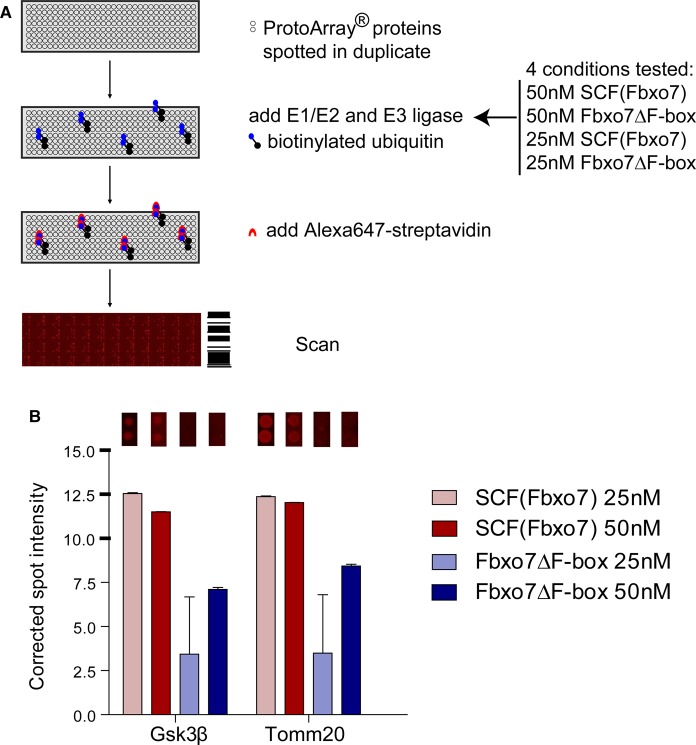
Identification of new Fbxo7 ubiquitinated substrates using protein arrays. (**A**) Schematic of the experimental design. Two concentrations of SCF^Fbxo7^ or Fbxo7(ΔF-box) protein were used for the ubiquitination of Protoarrays®. Unique substrates were grouped according to the Gene Ontology Cell Compartment or Biological Process through the use of Cytoscape (plugin ClueGO + CluePedia; Supplementary Table S4). (**B**) Both concentrations of the SCF^Fbxo7^, but not Fbxo7(ΔF-box) protein, promoted ubiquitination of Gsk3β and Tomm20. Quantification of the fluorescence intensity of individual spots.

We next optimized the SCF E3 ligase to perform a large-scale *in vitro* ubiquitination experiment using a protein microarray. The commercially available Protoarray® v5.0 (Invitrogen) contained over 9000 unique, full-length native proteins purified and spotted in duplicate under non-denaturing conditions onto a nitrocellulose slide. Ligase activity was first titrated, and a 50 nM concentration, which demonstrated robust auto-ubiquitination activity, was chosen for use on the array (Supplementary Figure S1C). Ligase (25 nM) was used in a replicate experiment, where more overall signal was seen on the array with the WT than the mutant ligase (Figure 2A,B). Overviews of the experimental design (Figure 2A) and the data analysis (Supplementary Figure S2, Materials and Methods) are provided. To increase the stringency of our screen, only proteins which met defined criteria for statistically significant intensities at both 25 and 50 nM of ligase were categorized as possible substrates. This analysis yielded 338 unique proteins as substrates of SCF^Fbxo7^ (Supplementary Table S5).

Candidate substrates were next grouped in accordance with their cellular compartment and their biological pathway using the Gene Onthology and KEGG databases for data enrichment (Supplementary Table S5) [39,40]. Forty-six candidate substrates were localized to the nuclear lumen, 39 to the cytosol, 13 to the ribosome, and 10 to the microtubule-organizing centre. This wide distribution pattern of candidate substrates is in agreement with the reported localization of Fbxo7 [10,22], and with the analyses of Fbxo7 ligases (Supplementary Table S4). Consistent with the presence of a mitochondrial-targeting sequence (MTS) at the N-terminus of Fbxo7, 29 candidates were localized to mitochondria, where Fbxo7 interacts with Parkin and PINK1 to mediate stress-induced mitophagy [21]. Such candidates represent potential Fbxo7 substrates that may be important for this process. In addition, although PINK1 is present on the Protoarray® (Supplementary Table S1), it was not ubiquitinated by SCF^Fbxo7^, suggesting that their reported interaction does not result in PINK1 ubiquitination. An analysis of the substrates using Enrichment by Biological Process indicated that Fbxo7 ubiquitinates multiple proteins that have an impact on ErbB2 (10 substrates), Wnt (10 substrates), and MAPK (13 substrates) signalling, ribosomes (9 substrates), axonal guidance (10 substrates), and pathways in cancer (16 substrates). We reported that Fbxo7 regulated cyclin D/Cdk6/p27 complexes which transformed cells, via an ubiquitin-independent mechanism [10], and in agreement with this, cyclin D3, Cdk6, and p27 were present on the Protoarray® v5.0 (Supplementary Table S1), but not ubiquitinated. Our dataset further indicates that SCF^Fbxo7^ also ubiquitinates proteins important in cancer progression. With regard to concordance with known SCF^Fbxo7^ substrates, cIAP-1, HURP, and NRAGE were not present on the protein arrays, and although TRAF2 was, it did not make the final list of substrates due to high levels of signal with the negative control Fbxo7(ΔF-box). We speculate that as TRAF2 is also an E3 ligase, it catalyzed auto-ubiquitination in the presence of the ubiquitin mix used in this experiment, hence the high background observed in the negative control.

These data demonstrate the robust ligase activity of the SCF^Fbxo7^ ligase and its potential to ubiquitinate a large number of proteins. Importantly, there was no overlap between the SCF^Fbxo7^ substrates with those identified in a previously reported SCF^Fbxo25^ Protoarray® screen [41], arguing that this methodology identifies specific candidate substrates for individual SCF-type E3 ligases.

### Gsk3β and Tomm20 were ubiquitinated *in vitro* and *in vivo* by SCF^Fbxo7^

**Figure 3.**
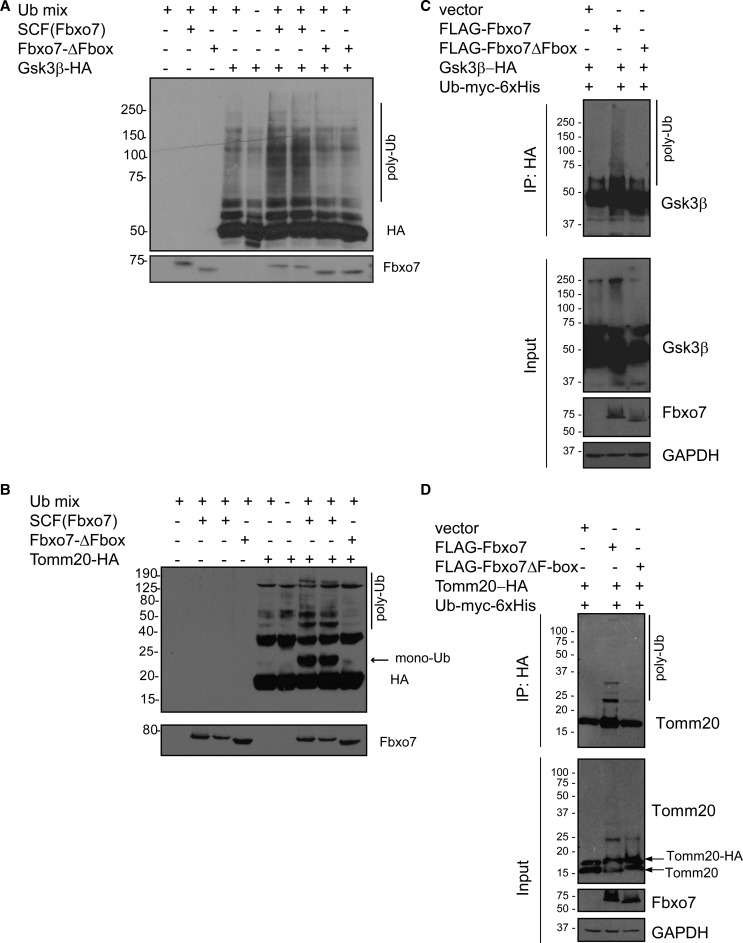
Fbxo7 promotes ubiquitination of Gsk3β and Tomm20 *in vitro* and in cells. *In vitro* ubiquitination reactions contained purified SCF^Fbxo7^ ligase or Fbxo7(ΔF-box) protein and ubiquitin mix (E1, UBE2D1, ubiquitin, and ATP) in combination with HA-tagged substrates Gsk3β (*n* = 2) (**A**) or Tomm20 (*n* = 2) (**B**). The proteins were resolved by SDS–PAGE, and immunoblots were performed with the indicated antibodies. (**C** and **D**) HEK293T cells were transfected with the indicated plasmids and the substrates were IP with anti-HA beads; Gsk3β (*n* = 3) (**C**) and Tomm20 (*n* = 3) (**D**). Proteins were separated by SDS–PAGE, and immunoblots were probed with antibodies to the substrates.

One caveat inherent in using protein arrays is that the full-length proteins spotted on the arrays are purified from insect cells in fusion with both His and GST tags, which may affect ubiquitination. Also, these proteins would lack the posited PTMs and/or cofactors, which may be needed for ligase recruitment within cells. We therefore wished to retest our findings from the arrays using independent methodologies. To take this forward, we selected Gsk3β, ranked 38th, and Tomm20, ranked 44th (Supplementary Table S5), because of the existing literature arguing that they act within pathways that are potentially involved in the pathobiology of PD, and because inhibitors of these pathways are being pursued as potential therapeutics [21,42–47]. Their ubiquitination signal on the arrays is shown in Figure 2B. We produced these potential substrates by *in vitro* transcription/translation in reticulocyte lysates using plasmids encoding C-terminally HA-tagged substrates, Gsk3β-HA or Tomm20-HA, and used these programmed lysates for *in vitro* ubiquitination assays (Figure 3A,B and Supplementary Figure S3A,C). We also used purified Gsk3β-HA or Tomm20-HA isolated by immunoprecipitation from cells and eluated with HA peptide as the substrates for *in vitro* ubiquitination assays (Supplementary Figure S3B,D). In the case of Gsk3β, a smear of higher MW bands was seen in crude reticulocyte lysates when no exogenous ligase was added to the reaction, suggesting that Gsk3β modification occurred in reticulocyte lysates (Figure 3A and Supplementary Figure S3A). However, there was an increased intensity of higher MW bands in the presence of WT SCF^Fbxo7^ ligase, which was dependent on the F-box domain (Figure 3A and Supplementary Figure S3A,B). For Tomm20, the addition of WT SCF^Fbxo7^ ligase promoted discrete laddering, suggesting mono- or multi-mono-ubiquitination, and was also dependent on the F-box domain of Fbxo7 (Figure 3B, arrows). In addition, higher MW bands, dependent on Fbxo7 ligase activity, were also detectable using antibodies to Tomm20 (Supplementary Figure S3C,D, arrows). Taken together, these data support the idea that SCF^Fbxo7^ catalyzed ubiquitination of these proteins.

To further test whether these candidate targets are substrates for SCF^Fbxo7^, we carried out ubiquitination assays in HEK293T cells co-transfected with plasmids encoding C-terminally HA-tagged substrates including Gsk3β-HA or Tomm20-HA and N-terminally FLAG-tagged Fbxo7 or Fbxo7(ΔF-box). Substrates were IP via their HA tag, and immunoblotted with antibodies to the protein (Gsk3β or Tomm20; Figure 3C,D). In the samples co-transfected with WT Fbxo7, a smear of higher MW proteins was seen for Gsk3β (Figure 3C), while discrete laddering was observed for Tomm20 (Figure 3D). Similar results were obtained when experiments were performed with endogenous ubiquitin (Supplementary Figure S3E,F). In addition, in *in vivo* ubiquitination assays performed with exogenous myc-tagged ubiquitin, Tomm20 showed discrete laddering, and both proteins showed robust smears of high-MW polyubiquitinated proteins over 100 kDa (Supplementary Figure S3G,H). These data support the idea that Fbxo7 promoted ubiquitination of both Gsk3β and Tomm20, and taken together, with the *in vitro* ubiquitination assays indicate that these two candidates identified on the protein arrays are ubiquitinated substrates of SCF^Fbxo7^.

### The N-terminus of Fbxo7 can mediate an interaction with Gsk3β

**Figure 4.**
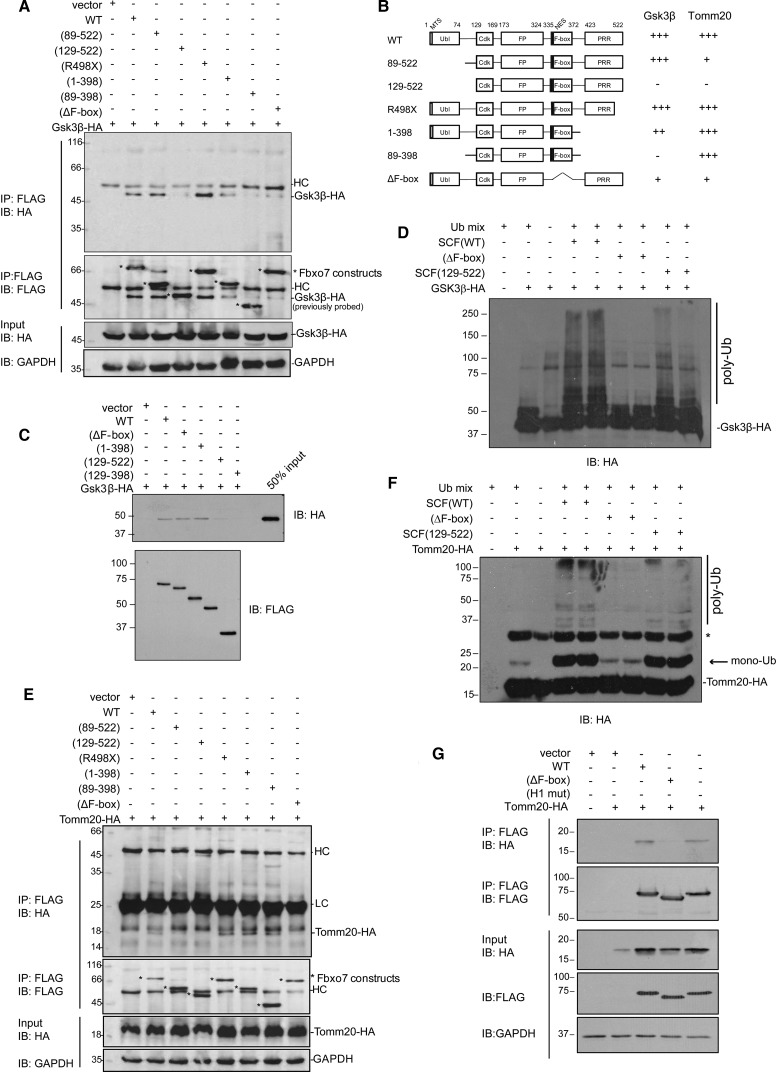
The N-terminus of Fbxo7 mediates its interaction with Gsk3β. (**A**) HEK293T cells were transfected with the indicated FLAG-Fbxo7 plasmids and Gsk3β-HA and the lysates were IP with anti-FLAG beads. Proteins were resolved by SDS–PAGE, and immunoblots were probed with the indicated antibodies. (**B**) Summary of the interaction mapping for Gsk3β and Tomm20 with various Fbxo7 constructs. (**C**) *In vitro* binding assays using immunopurified and eluted Gsk3β-HA protein added to various Fbxo7 proteins lacking N- and C-terminal domains immobilized on anti-FLAG antibodies on agarose. (**D**) *In vitro* ubiquitination assay using an SCF^Fbxo7(129–522)^ ligase lacking the N-terminus against immunopurified and eluted Gsk3β-HA protein as the substrate. (**E**) HEK293T cells were transfected with the indicated FLAG-Fbxo7 plasmids and Tomm20-HA and the lysates were IP with anti-FLAG beads, as in (**A**). (**F**) *In vitro* ubiquitination assay using an SCF^Fbxo7(129–522)^ ligase lacking the N-terminus against immunopurified and eluted Tomm20-HA protein as the substrate. (**G**) *In vivo* co-immunoprecipitation assays conducted as in (**E**).

To determine the region within Fbxo7 required for interacting with these substrates in cells, co-immunoprecipitation assays were conducted. HEK293T cells were co-transfected with plasmids encoding C-terminally HA-tagged Gsk3β and various N-terminally FLAG-tagged Fbxo7 constructs: WT or Fbxo7 constructs wherein the Ubl domain (1–88), a linker region (89–128), the PRR (399–522), or the last 24 aa at the C-terminus (R498X) was deleted either singly or in combination. The R498X mutation is a naturally occurring pathogenic mutation causing an early-onset PD. For Gsk3β, the loss of either the C-terminal 24 aa (R498X) or the PRR (1–398) did not substantially affect binding (Figure 4A). Loss of the N-terminal Ubl domain, as tested with the 89–522 construct, also did not affect Fbxo7 interaction with Gsk3β. However, loss of both the Ubl domain and the linker, as seen with a 129–522 construct, ablated their interaction. At the Fbxo7 C-terminus, loss of the PRR alone, 1–398, weakened binding, but the additional loss of the Ubl domain, as tested with the 89–398 construct, ablated Gsk3β binding altogether. These data suggest that the PRR, together with the Ubl/linker sequences, contributes to the interaction of Fbxo7 with Gsk3β in cells, but is not sufficient to mediate binding (Figure 4A). These experiments indicate multiple binding sites and/or a bipartite interaction for Gsk3β with Fbxo7. These interaction experiments are summarized in Figure 4B. To further validate the binding sites of Gsk3β on Fbxo7, we performed *in vitro* binding assays using FLAG-Fbxo7 deletion mutants immunopurified from cells and immobilized on agarose, in binding assays with purified Gsk3β (Figure 4C). In support of the *in vivo* co-immunoprecipitation studies, Fbxo7 constructs lacking the first 129 amino acids, deleting the Ubl and linker of Fbxo7 were unable to interact with Gsk3β. Fbxo7 1–398 lacking only the PRR could still bind to Gsk3β, as could ΔF-box in this *in vitro* setting. To test this further, we created a ligase lacking the N-terminal 128 amino acids for its ability to ubiquitinate Gsk3β. We found that SCF^Fbxo7(129–522)^ was an E3 ubiquitin ligase with equivalent activity to SCF^Fbxo7^ (Supplementary Figure S4A), which showed reduced activity against Gsk3β tested in *in vitro* ubiquitination assays (Figure 4D). These data argue for the N-terminus of Fbxo7 being the domain through which Gsk3β can be recruited for ubiquitination.

Similar experiments to map the interaction of Fbxo7 with Tomm20 were conducted in cells (Figure 4E) and by *in vitro* binding assays, although the latter were unsuccessful. In cells, the interaction of Tomm20 with Fbxo7 was substantially weakened when the N-terminus was deleted, see 89–522 and 129–522, suggesting that amino acids 1–128 also mediated the interaction of Fbxo7 and Tomm20. However, unlike Gsk3β, which is reported to localize in the cytosol, nucleus, and mitochondria, Tomm20 is part of a tranlocation complex and resident in the outer membrane of mitochondria. Therefore, an alternative explaination for this loss of binding could be the deletion of the MTS of Fbxo7, which lies at the start of isoform 1 (Figure 4B) and is essential for its recruitment to mitochondria [21]. To test this directly, an *in vitro* ubiquitination assay with SCF^Fbxo7(129–522)^ against Tomm20 was performed and showed that the ligase lacking the N-terminus of Fbxo7 was still able to mono-ubiquitinate Tomm20, and that there was only minor loss of ubiqutinated higher MW species (Figure 4F). These data argue that other sites downstream of amino acid 128 in Fbxo7 bind to Tomm20. Deletion of the PRR at the C-terminus of the Fbxo7 (1–398) did not compromise binding to Tomm20, but suprisingly, deletion of the PRR restored Tomm20 binding to Fbxo7 lacking the MTS/Ubl domain (Figure 4, compare 89–522 binding with 89–398). Potential reasons for this include that the PRR inhibits Fbxo7 interaction with Tomm20 or the smaller 89–398 construct can localize to the mitochondria. Combined, these data indicate that sequences between 89 and 398 of Fbxo7 can bind to Tomm20 in cells. These sequences include the F-box domain (335–372), and we also noted a diminished interaction of Tomm20 with an Fbxo7 construct lacking the F-box domain similar to Gsk3β (Figure 4A,E). However, this was likely to be due to a change in the localization of Fbxo7 as a result of a deletion of an NES embedded within the first helix of the F-box domain [22]. We previously demonstrated that the NES of Fbxo7 is important for its cell cycle-dependent cytoplasmic/nuclear shuttling, and if mutated, Fbxo7 becomes a predominantly nuclear protein. To test whether the ΔF-box mutant does not interact with Tomm20 because of its localization to the nucleus, we utilized an Fbxo7 quadruple point mutant, called H1, which mutates the F-box domain such that it does not bind to Skp1 but leaves a functional NES. The H1 mutant localizes similarly to WT, predominantly cytoplasmic with nuclear shuttling [22]. We directly compared the H1 mutant with the ΔF-box mutant for their interaction with Tomm20 using co-immunoprecipitation studies, and found the H1 mutant can bind to Tomm20, whereas the ΔF-box mutant does not (Figure 4G), supporting the nuclear localization of the NES/ΔF-box mutant is the likely cause for the lack of interaction with Tomm20. Both mutants do not bind to Skp1, which also indicates that Fbxo7 can bind to Tomm20 independent of it being part of an E3 ligase.

### SCF^Fbxo7^ promotes non-degradative chain formation on Gsk3β and Tomm20

**Figure 5.**
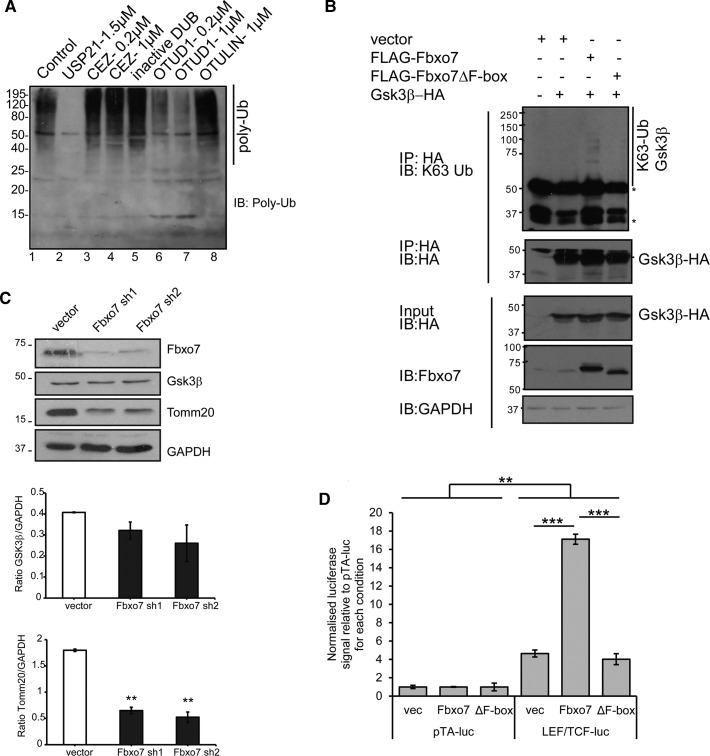

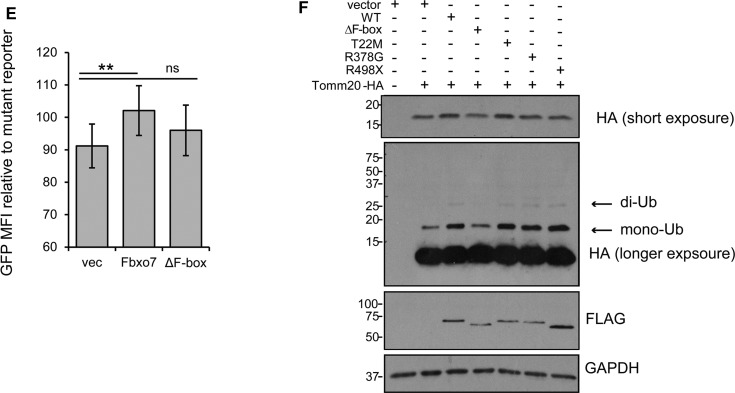
Analysis of ubiquitin chain linkages catalyzed on SCF^Fbxo7^ substrates Gsk3β and Tomm20. (**A**) UbiCRest analyses using DUBs for polyubiquitinated Gsk3β purified from cells (*n* = 2). The non-specific DUB (USP21) cleaved all ubiquitin chains from polyubiquitinated Gsk3β, and K63-specific DUB, OTUD1, showed concentration-dependent activity against polyubiquitinated protein. (**B**) Immunoprecipitation of Gsk3β from cells and detecting the presence of K63 polyubiquitin chains using a K63-specific antibody (*n* = 2). Asterisk indicates heavy or light chains. (**C**) Effect of reducing Fbxo7 expression on expression of Gsk3β and Tomm20. HEK293T cell lines expressing two independent shRNAs for Fbxo7 (Fbxo7 sh1 and sh2) or vector control were used. Immunoblotting with the indicated antibodies was performed and protein levels were determined relative to GAPDH. The ratio of the densitometry value for each substrate relative to GAPDH is graphed (*n* = 3). (**D**) U2OS cells were co-transfected with an empty luciferase vector (pTA-luc) or a β-catenin-responsive TCF/LEF luciferase reporter, along with a Renilla luciferase reporter as a transfection control. Cells were also co-transfected with an empty vector (vec), Fbxo7 or Fbxo7(ΔF-box). After 48 h, cells were harvested and luciferase expression was assayed in quintuplicate from cell lysates using the Dual Glo kit according to the manufacturer's instructions (Promega), and luciferase levels were normalized to Renilla levels and expressed relative to pTA-luc control levels (*n* = 3). (**E**) U2OS cells were transfected with MAPK-Gsk3β-GFP or MAPK-Gsk3βmut-GFP reporters with empty vector, Fbxo7 or Fbxo7(ΔF-box), and GFP expression was determined by flow cytometry. GFP mean fluorescence intensity (MFI) was expressed as a percentage of MAPK-Gsk3βmut-GFP levels (*n* = 6). (**F**) U2OS cells were co-transfected with Tomm20-HA and the indicated plasmids bearing Fbxo7 WT or PD-associated mutant alleles, and immunoblots on total cell lysates were performed for the transfected proteins as indicated, and GAPDH used as a loading control. ***P-value is <0.001, **P-value is <0.01.

The type of polyubiquitin chain linkages catalyzed by an E3 ligase on a substrate induces different functional consequences [48]. To investigate the types of ubiquitin chains ligated by SCF^Fbxo7^ onto Gsk3β, we carried out UbiCRest analyses by using DUBs as described in ref. [34]. The source of the ubiquitinated protein used in DUB assays was obtained by immunoprecipitating Gsk3β-HA from cells also transfected with Fbxo7 as in Figure 3A, where Fbxo7 expression enhanced a smear of ubiquitination. Ubiquitinated Gsk3β was subjected to cleavage using a panel of DUBs, including as a positive control, USP21, a non-specific DUB which cleaves all types of ubiquitin chains (Figure 5A). Only increasing amounts of the K63-specific OTUD1 concentration diminished the intensity of the smear of polyubiquitinated Gsk3β (Figure 5A, lanes 6 and 7), indicating that Fbxo7 catalyzed predominantly K63-linked ubiquitin chains. To independently test for the presence of Fbxo7-enhanced K63 chains on Gsk3β, HEK293T cells were co-transfected with Gsk3β-HA and Fbxo7 or ΔF-box domain. Immunoprecipitates of Gsk3β-HA were probed for the presence of K63 chains, and these were detected when Fbxo7, but not ΔF-box, was overexpressed (Figure 5B).

To test the effect of SCF^Fbxo7^ ubiquitination on Gsk3β and Tomm20, their total levels were assayed by immunoblotting of HEK293T cell lysates where constitutive knockdown (KD) of Fbxo7 expression was achieved by stable expression of two independent short hairpin miRNA constructs. Consistent with the identification of mainly K63 polyubiquitination by SCF^Fbxo7^ from UbiCRest analyses, reducing Fbxo7 expression did not alter Gsk3β levels (Figure 5C). These results were also confirmed by similar experiments in SHSY-5Y KD cells (data not shown) and in experiments where cells were transiently transfected with dsRNA targeting Fbxo7 in U2OS cells (Supplementary Figure S4B). These findings suggest that ubiquitination may modulate its localization or function. Gsk3β is an upstream regulator of β-catenin, which has roles both in transcription through activation of TCF/LEF-binding sites and in adhesion through its binding to cadherins. We first tested whether Fbxo7 affected transcriptional activation using a TCF/LEF luciferase reporter, which is responsive to β-catenin, in U2OS cells. We found that the overexpression of Fbxo7 increased transactivation from this reporter, and this was dependent on the F-box domain (Figure 5D). We also show that inhibition of Gsk3β upon LiCl treatment strongly activates the TCF/LEF reporter, and no further induction of reporter activity is seen with Fbxo7 expression, arguing for its effect being via Gsk3β inhibition (Supplementary Figure S4C). We also tested whether Gsk3β localization was changed as a result of Fbxo7 expression, but no significant differences were observed by immunofluorescence assays, neither when Fbxo7 was overexpressed nor when its levels were reduced (Supplementary Figure S4D,E). Gsk3β levels were also not significantly altered by Fbxo7 overexpression (Supplementary Figure S4F). To determine if Fbxo7 affected Gsk3β activity, we utilized a GFP reporter with a degron that is sensitive to levels of Gsk3β activity [37]. For comparison, a GFP reporter with alanine substitutions of the Gsk3β phosphoacceptors in the degron was used. The co-expression of Fbxo7, but not the ΔF-box mutant, with the GFP reporter significantly increased the mean fluorescence intensity of the cells compared with vector only expressing cells, indicating a decrease in Gsk3β kinase activity (Figure 5E). Taken together, these data argue for a repressive effect of SCF^Fbxo7^ ubiquitination on Gsk3β activity.

In the case of Tomm20, there was a significant decrease in its endogenous levels when Fbxo7 was knocked down constitutively or by transient transfection of siRNA (Figure 5C and Supplementary Figure S4B), suggesting that Fbxo7 stabilizes Tomm20 levels. Conversely, a small and reproducible 50% increase was observed in endogenous Tomm20 levels when Fbxo7 was overexpressed, (Supplementary Figure S4F). These data indicate a direct correlation between endogenous Fbxo7 and Tomm20 expression levels. However, results in Figure 4G argue that stabilization of Tomm20 occurred independently of Fbxo7 being part of an SCF complex. As can be seen in Figure 4G, overexpression of WT Fbxo7 and the H1 mutant both caused increased Tomm20-HA levels (Input, IB: HA, lanes 3 and 5), even though H1 does not recruit Skp1. These data suggest that the stabilization of Tomm20 does not require ubiquitination by SCF^Fbxo7^.

We also tested whether the mutants in Fbxo7 associated with early-onset PD affected the ubiquitination of Tomm20 by co-transfecting cells with the mutant Fbxo7 alleles (T22M, R378G, and R498X) and Tomm20-HA (Figure 5F). The presence the higher MW mono- and di-ubiquitinated Tomm20 were not altered by co-transfection of mutant alleles of Fbxo7 when compared with WT Fbxo7. Importantly, as the T22M Fbxo7 mutant cannot bind to Parkin, these data strongly argue that the ubiquitination detected was not due to Parkin-mediated ubiquitination, but rather mediated by Fbxo7. These data indicate that mutant Fbxo7 alleles were capable of enhancing Tomm20 ubiquitination, and suggest that defects in Tomm20 ubiquitination do not contribute to the aetiology of the Fbxo7 cases of early-onset PD.

## Discussion

In a study of the Cullin interactome using MS and where the abundance of the cullin-associated proteins was calculated based on peptide recovery, Fbxo7 was the fifth most abundant F-box protein identified (behind Skp2, Fbxl18, Fbxo21, and Fbxo22), suggesting that SCF^Fbxo7^ is a stable, abundant E3 ligase in a modified 293 cell line [49]. In agreement with this prediction, we found that the purified SCF^Fbxo7^ ligase from HEK293T cells was indeed abundant, stable and robustly active when used for *in vitro* assays. A total of 338 unique proteins, or about 3.6% of the proteins on the array, were defined as putative substrates of SCF^Fbxo7^ ubiquitination using a cell-independent methodology to obtain a global view of its activity. Approximately 123 (36%) of the Fbxo7 ubiquinome are listed as ubiquitinated proteins in either HEK293T and/or U2OS cells [50,51], with 17% of this subset affected by treatment with a proteasomal inhibitor MG132. In comparison, the number of substrates identified in the present study is about four times those for SCF^Fbxo25^ and SMURF1 where 89 and 75 substrates, respectively, were identified using a similar experimental approach [33,41]. The substrates for SCF^Fbxo7^ do not overlap with these other screens, arguing for the specificity of these ligases in comparable settings. We hypothesize that within specific cell types, SCF^Fbxo7^ activity and the chain linkages assembled will be refined and dictated by the expression levels of Fbxo7, the abundance and potential PTM of its substrates, and the E2 ligases with which it engages.

An enrichment analysis of KEGG pathways revealed that several proteins directly involved in the Wnt signaling pathway, including Csnk1E, Gsk3β, Prickle2, and Nkd2, were ubiquitinated by SCF^Fbxo7^ on the Protoarray®. Gsk3β phosphorylates proteins like β-catenin, Snail, and Smad, which creates a degron for E3 ligases SCF^β-TRCP^ and SMURF1 that ubiquitinates them to promote their proteasomal degradation [52–55]. The deregulation of this pathway is associated with several types of cancer [56,57]. In addition to its function in the Wnt pathway, Gsk3β phosphorylates a large number of proteins located throughout the cell and is involved in many cellular processes such as cell proliferation, differentiation, microtubule dynamics, cell cycle, and apoptosis [58]. With such pleiotropic function, Gsk3β is linked with many different diseases including diabetes, cancer, Alzheimer's disease, osteoporosis, and cardiac hypertrophy, and also with PD [59]. Investigations into the role of Gsk3β in PD have uncovered many associations including a high level of kinase activity within the striatum and also the finding that a phosphorylated form of Gsk3β is found surrounding Lewy bodies, which may stem from the fact that Gsk3β can directly phosphorylate α-synuclein [60–62]. Gsk3β is also an interesting therapeutic target, and several small-molecule inhibitors have already been described [63]. We find that SCF^Fbxo7^ can ubiquitinate Gsk3β and promote K63 linkages. Fbxo7 did not affect Gsk3β endogenous levels or localization, but instead repressed its activity leading to increased expression of a Gsk3β-sensitive GFP reporter and increased β-catenin transactivation in cells. Our data place Fbxo7 in the complex regulatory landscape of Gsk3β function and activity, and indicate that the consequences of Gsk3β ubiquitination by SCF^Fbxo7^ warrant further investigation in the context of neurodegenerative PD model systems.

Fbxo7 has a reported role in mitophagy through its direct association with two other PD associated proteins, PINK1/*PARK6* and Parkin/*PARK2* [21]. Upon depolarization, Tomm20 is one of the mitochondrial proteins that is ubiquitinated by Parkin [64,65]. Tomm20 is a core component of the mitochondrial translocase complex, and its overexpression alone can promote mitophagy [44]. We demonstrate here that under normal conditions, WT Fbxo7 and the mutant alleles of Fbxo7 stabilized its levels, which did not depend on SCF formation, and promoted Tomm20 mono-, multi-mono-, or di-ubiquitination. The overexpression of human Fbxo7, but not the PD-associated alleles, rescues the phenotypes of *parkin* loss in a *Drosophila* model of neurodegeneration [21]. However, the WT and mutant alleles tested have a similar ability to ubiquitinate Tomm20, indicating that improper ubiquitination of Tomm20 by Fbxo7 is unlikely to be the pathological deficiency. These included a T22M mutant of Fbxo7, which cannot recruit Parkin, indicating Parkin was not catalyzing Tomm20 ubiquitination in these experiments. An enrichment analysis of the substrates by GO Cellular Compartment revealed a further 29 mitochondrial proteins, which may indicate a more general role of Fbxo7 in mitochondrial biology, and suggests that Fbxo7 may affect other activities through ubiquitination of substrates, like ATP5C1, CHCHD2, and MTIF3, mitochondrial translation initiation factor 3 (Supplementary Table S5). We note that the only reported polymorphism within *MTIF3*, rs7669, has been reported to show a significant association with risk of PD [66], and CHCHD2 also has been reported to be associated with cases of autosomal dominant PD [67–69]. These substrates indicate a connected network among the genes mutated in familial PD data and are an area for future study.

In summary, we identified 338 new targets of SCF^Fbxo7^ using a high-throughput, cell-independent proteomic approach and validated Gsk3β and Tomm20 as new substrates, and argue against defective regulation of Tomm20 by Fbxo7 as an underlying mechanism in PD. On the basis of our findings, we predict that Fbxo7 will impact on multiple biological functions, and this potentially explains why this F-box protein is of clinical importance in pathologies affecting many different tissue and cell types. Our dataset opens new perspectives and avenues for investigation in determining the role of SCF^Fbxo7^ ligase activity in the biology of human diseases, including PD and cancer.

## Abbreviations

AB, assay buffer; BMP, bone morphogenic protein; BSA, bovine serum albumin; c-IAP1, inhibitor of apoptosis protein 1; DDA, data-dependent acquisition; DTT, dithiothreitol; DUBs, deubiquitinating enzymes; E1, ubiquitin-activating enzyme; E2, ubiquitin-carrier enzyme; FC, fold change; Gsk3β, glycogen synthase kinase 3β; HA, human influenza hemagglutinin; HRP, horseradish peroxidase; HURP, hepatoma up-regulated protein; IVT, *in vitro* transcription/translation; LB, lysis buffer; LEF/TCF, lymphoid enhancer factor/T-cell factor; LQT, linear trap quadrupole; MTS, mitochondrial-targeting sequence; MW, molecular weight; NB, NETN lysis buffer; NES, nuclear export signal; NRAGE, neurotrophin receptor-interacting MAGE; PD, Parkinson's disease; PTMs, post-translational modifications; SCF, Skp1-Cul1-F box protein; SNP, single nucleotide polymorphism; Tomm20, translocase of outer mitochondrial membrane 20; TRAF2, TNF receptor-associated factor 2; UbiCRest, ubiquitin chain restriction; WT, wild type.

## Author Contribution

F.R.T., S.J.R., S.P.P., T.E.T.M., G.Z., and T.K. conducted experiments and analyzed data. D.K. and H.L. designed experiments, analyzed data and wrote the manuscript. All authors read and edited the manuscript.

## Funding

F.R.T. was funded by a BEPE-FAPESP Fellowship [2010/16464-8, 2012/09241-8]. S.J.R. and H.L. are funded by the Biotechnology and Biological Science Research Council [BB/J007846/1]. D.K. is funded by the European Research Council [309756], Medical Research Council [U105192732] and the Lister Institute for Preventive Medicine. T.E.T.M. was funded by the Marie Curie ITN ‘UPStream’.
